# Lunatic Asylums in the North-Western Provinces of India and Oudh

**Published:** 1902-11-08

**Authors:** 


### The Institutional Workshop.

### LUNATIC ASYLUMS IN THE NORTH-WESTERN PROVINCES OF INDIA AND OUDH.

The population in these asylums for the year 1900 practically remained stationary, there being a difference of three only between January 1st and December 31st. The total number of lunatics for which the asylums provide accommodation is 1,260, but there were never more than 1,124 at any given time during the year. In the Benares and Lucknow asylums, however, the limit of accommodation for male inmates was reached, and consequently the admission of criminal lunatics was temporarily stopped. Government has now sanctioned the proposal that the buildings already erected at Agra in connection with the project for an amalgamated asylum be made ready at once for occupation by European and Eurasian insanes, of whom there are at present 16 confined in the North-Western Province Institutions. The deaths in the asylums rose from 72 in 1899 to 82 in 1900. The death rate in the Lucknow Asylum showed no decrease, and the health in the Bareilly Asylum was unsatisfactory. Cholera made its appearance in September and attacked 12 persons, of whom six died. It was found impossible to trace the means by which the disease was introduced, but it was prevailing epidemically at the time in the city and district of Bareilly, and no doubt it was brought in either in the supplies or through warders whose families became affected. The chief cause of mortality, however, was phthisis. This complaint is, according to statistics, specially liable to attack the insane in all countries, and it is most decidedly prevalent in the Bareilly Asylum, about one-third of the deaths being due to it. There is no doubt that it is conveyed by the ordinary means of infection, and until insane patients, affected by consumption, can be isolated the disease must spread. As there were at least a dozen cases of phthisis under treatment at the end of 1900, the statistics for the following year will not probably show a less serious death rate. Of those who died only four were in good health upon admission, whilst the health of seven was certified as "indifferent" and that of 10 more was "bad." At the Lucknow Asylums there were 25 deaths as against 21 in the previous year. Fifteen of these patients were in a weak and debilitated condition when they were admitted, and no hopes were entertained of their ultimate recovery; they were only kept alive by constant care and attention. Otherwise, the number of admissions to hospital was considerably less than in the previous year. It is a matter of congratulation that the cause of the insanity has been ascertained in a far larger number of cases than before, showing a greater research into the past history of the patient, and that more trouble has been taken in the matter by the medical superintendent. Owing to high prices and a large population, the cost of maintenance of the lunatics during the year amount to Rs.81,000, or an increased expenditure of Rs.11,000. On the other hand, the sum contributed by the friends of European and Eurasian lunatics (Rs.2,300) shows a considerable advance on the contributions of the previous year, and this reduced the cost of these lunatics to the Government. The amount realised by asylum industries fell to a little over Rs.3,000 mainly owing to the Bareilly Asylum, which formerly showed a large profit in connection with the dairy farm, showing a loss. There was heavy mortality amongst the cattle, 73 animals having died, but from what cause is not known. Poultry farming is not found to pay, so that all the poultry has been sold off. There were embezzlements during 1900 in the feed of the dairy cattle. A change has been made in the staff, and the retention of the dairy farm as part of the asylum is still under consideration. The most important work carried out during the year was the introduction of the municipal filtered water supply into the Benares Asylum. The buildings for European and Eurasian lunatics now completed at Agra will eventually form part of a central lunatic asylum at that place, under the charge of a full-time superintendent, to whose appointment the sanction of the Secretary of State has been obtained. The plans of the other new buildings required for this asylum were discussed in view of modern requirements by a conference held at Agra during 1900, and the Government have already passed orders upon recommendations then made.

